# In-transit development of color abnormalities in turkey breast meat during winter season

**DOI:** 10.1186/s40781-017-0157-1

**Published:** 2018-01-22

**Authors:** Rafael H. Carvalho, Danielle C. B. Honorato, Paulo D. Guarnieri, Adriana L. Soares, Mayka R. Pedrão, Alexandre Oba, Fernanda G. Paião, Elza I. Ida, Massami Shimokomaki

**Affiliations:** 1 Graduate Program in Animal Science, Department of Veterinary Preventive Medicine, Londrina State University, CEP 86010-951, Londrina, PR PO Box 6001 Brazil; 2 Graduate Program in Food Science, Department of Food Science and Technology, Londrina State University, Londrina, PR Brazil; 3 Graduate Program in Food Science, Sao Paulo University, Sao Paulo, SP Brazil; 4 Professional Master Program, Paraná Federal Technological University in Londrina, Campus Londrina, Londrina, PR Brazil

**Keywords:** Animal welfare, Commercial slaughterhouse, DFD-like, PSE-like, Winter climate

## Abstract

**Background:**

The poultry industry suffers losses from problems as pale, soft and exudative (PSE), and dark, firm and dry (DFD) meat can develop in meat as a result of short- and long-term stress, respectively. These abnormalities are impacted by pre-slaughter animal welfare.

**Methods:**

This work evaluated the effects of open vehicle container microclimate, throughout the 38 ± 10 km journey from the farm to the slaughterhouse, on commercially turkey transported during the Brazilian winter season. The journey was initiated immediately after water bath in truck fitted with portable Kestrel anemometers to measure air ventilation, relative humidity, temperature and ventilation.

**Results:**

The inferior compartments of the middle and rear truck regions showed highest temperature and relative humidity, and lower air ventilation. In addition, the superior compartments of the front truck regions presented lower temperature and wind chill, and highest air ventilation. The breast meat samples from animals located at the inferior compartments of the middle and rear truck regions and subjected to with water bath (WiB) treatment presented highest DFD-like and had lowest PSE-like meat incidence than those from animals located at other compartments within the container. Lower incidence of PSE-like meat was observed in birds without water bath (WoB).

**Conclusions:**

Assessment on turkeys transported under Brazilian southern winter conditions revealed that breast meat quality can be affected by relative humidity, air ventilation, temperature, and transport under subtropical conditions promoting color abnormalities and the formation of simultaneously PSE-like and DFD-like meat.

## Background

The poultry industry is subjected to several detrimental factors that impact meat quality [1–3]. During transport from the farm to the slaughter plant, birds are subjected to stressors that compromise their welfare, affecting muscle metabolism resulting in deterioration of meat quality [2, 4–6]. These in-transit stress factors are primarily related to variability of transport container microclimates [5, 7, 8].

Previous experiments have established in transit the existence of air ventilation deviations that impact animal welfare [9–11]. Kettewell and Mitchell [12] conducted a three-dimensional characterization of the environmental conditions inside the cargo hold of commercial trucks loaded with chickens and reported great regional disparities of temperature, humidity, and ventilation within loads.

Several reports indicated that there is a thermal core in which thermal load and relative humidity are higher and that this core corresponds to low-ventilation regions within the loaded truck container [9–11, 13]. However, these studies were carried out within temperate regions. Experimental research is scarce within tropical climate zones that experience intense daily temperature fluctuations [1, 3, 11, 13, 14].

Previous reports have described the correlation of bird management practices, both at the farm and in transit to commercial slaughterhouses, on the prevalence of pale, soft and exudative (PSE-like) meat under summer conditions [7, 11, 15]. The stress promoted by longer transit journey caused depletion of muscle glycogen resulting in higher *postmortem* muscle pH [16, 17], which correlated with lower lightness (L*) value [5]. While PSE meat in poultry has been an economical matter of concern world widely, USA [18], Canada [19], Italy [20], Brazil [8], China [1], few reports deal with DFD meat [16, 17, 21].

The aim of this work was to evaluate the effect of current farm-to-slaughterhouse cargo transport practices during the winter season on turkey welfare and meat quality.

## Methods

### Experimental designed and statistical analysis

The experimental design was randomized into a 6 × 2 factorial arrangement (truck position × water bath treatment) with 6 positions: superior front (SF), inferior front (IF), superior middle (SM), inferior middle (IM), superior rear (SR) and inferior rear (IR) and two water bath treatments: with water bath (WiB) or without water bath (WoB), providing for 12 treatments, with 8 replicates for each treatment. WiB or WoB were carried out for 5 min just before leaving the farm. The data were analyzed using ANOVA and factorial analysis methods in the SPSS software. Meaningful comparisons were generated using Tukey’s test (probability less than 0.01 was considered significant). For incidences of PSE-like meat, the binary variation (1 and 0) was used, where 1 denoted PSE-like meat and 0 denoted normal meat. For incidences of dark, firm and dry (DFD-like) meat, the binary variation (1 and 0) was used, where 1 denoted DFD-like meat and 0 denoted normal meat.

### Animals

**Fig. 1:**
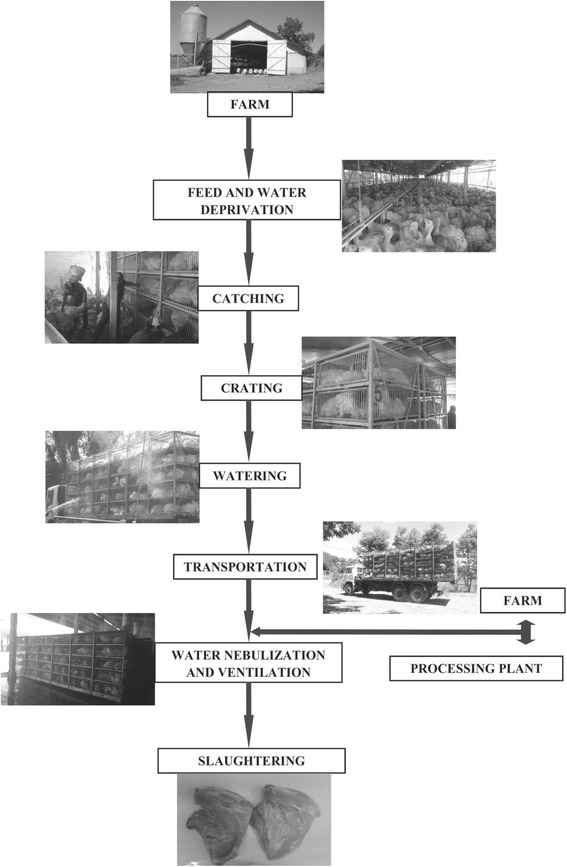
Turkey handling sequence for transportation from the farm to the processing plant

This study was conducted during Brazilian winter months between May 2015 and August 2015 in a commercial plant in Chapecó city area (Latitude: 27° 05′ 47" S; Longitude: 52° 37' 06" W; Altitude: 674 m), Santa Catarina State, within the southern region of Brazil. All sampling days had similar weather conditions: temperature (3 °C - 7 °C) and relative humidity (45% - 55%). The transportation conditions and activities from the farm to the turkey commercial processing plant are illustrated in the flow chart shown in Fig. 1.

The weather conditions in this region were characterized by the minimum and maximum temperatures of −5 °C and 26 °C, respectively, with relative humidity (RH) variations from 39 to 65% throughout the 73 days of sampling, as measured using a Kestrel 4000 instrument (Nielsen-Kellerman, Boothwyn, PA, USA). The birds were males of Nicholas 700 lineage grown under regular acclimatized aviaries to an age of 140 days and an average live weight of 18 ± 2 kg. The feed was removed 9 to 12 h before slaughter, and water was provided ad libitum. The animals were manually placed into crates at a density of 8 birds (98 ± 2 kg/m^2^) each and installed within the truck open container. The average catching and loading time was 24 min and after that the birds were subject or not to the water bath treatment for approximately 5 min. The water bath system consisted of 180 spray-heads; each with a diameter of 50 mm. The water temperature was between 8 and 10 °C (environment) and the total rate was 90–95 m^3^/h. Birds were handled in accordance with the principles and procedures outlined by the Londrina State University Animal Care and Use Ethical Committee (167/2015).

### Truck container microenvironment assessment

**Fig. 2:**
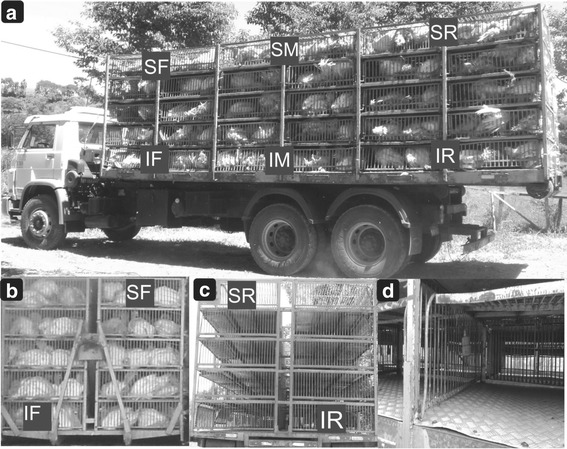
**a**) Photograph of the truck container side loaded with turkeys showing the strategic locations of the data-loggers: SF: Superior Front, IF: Inferior Front, SM: Superior Middle, IM: Inferior Middle (IM), SR: Superior Rear and IR: Inferior Rear. **b**) Photograph of the truck container front-view showing the positions of the columns and the data-loggers (dark gray). **c**) Photograph of the truck container rear-view showing the positions of the columns and the data-loggers (dark gray). **d**) Photograph showing the positions of the data-loggers in the transport cage

The truck had dimensions of 9 m long, 2.3 m wide and 2.5 m high. Six portable weather meter devices with bidirectional Kestrel anemometers and data logging capability were set to take measurements at 30-s intervals during each journey, as described in Spurio et al. [11], with minor modifications. Instruments were fixed and oriented in the direction of highest velocity, all devices were calibrated for relative humidity, temperature and ventilation before the experiment. The devices were installed laterally in cages (Fig. 2D) at the front, middle and rear truck container regions on the second, fourth and sixth columns, respectively, at a distance of 1.5, 4.5 and 7.5 m from the truck cab front in vertical duplicates: one data logger was located at the first box and the second was at the fifth box, respectively, at 0.25 m and 2.25 m above the truck base. The devices were installed laterally in cages (Fig. 2D) at the SF, IF, SM, IM, SR and IR of the truck container compartments and at positions between columns 1 and 2, 3 and 4, and 5 and 6, as shown by the gray rectangles in Fig. 2. Ambient temperature and relative humidity were measured at three different points: at the beginning of transport, arrival at the slaughterhouse plant and during holding time.

The relative humidity (RH), air ventilation (AV), and temperature (T) values were determined as in Spurio et al. [11], and wind chill (WC) values were simultaneously measured allowing for a representative analysis of the heterogeneous distribution of the thermal microenvironment within the loaded vehicle. WC, or apparent temperature, is an index that combines air temperature and air ventilation to measure the perceived equivalent corporal temperature [22]. RH, AV, T, and WC were automatically obtained from the 6 data loggers and downloaded to a computer.

### Transport and slaughter

A total of 32 transport journeys was evaluated, either with bath (WiB) (*n* = 8) or without bath (WoB) application (*n* = 8). These processes of birds harvesting and loading were only started when the ambient temperature has reached the temperature of 5 °C. The animals were grown on 8 turkey farms under a cooperative system and subsequently transported over a distance of 38 ± 10 km for a journey that took approximately 95 ± 20 min. Upon arrival at the slaughterhouse facilities, all birds were placed in a holding area under water mist and ventilation for approximately 70 min before slaughtering. The animals were sacrificed according to standard industry practices, which consisted of hanging, electrically stunning, bleeding, scalding, defeathering, evisceration, cooling the carcass through a tunnel of cold air (6 °C for 6 h) and deboning [8]. Subsequently, the breast meat samples (*Pectoralis major*) were collected and refrigerated at 4 °C for 24 h prior to analysis. Classification of meat as normal, PSE-like [8], and DFD-like [23] was performed by measurement of color and pH values.

### PSE-like and DFD-like meat measurement

Samples of breast fillets (*n* = 1344) (*Pectoralis major*) were collected 84 animals per transport load (*n* = 16)]. The meat samples were classified as PSE-like, normal, and DFD-like meat (Table 1) by the pH and lightness (L*) values [8, 23].

**Table 1:** Classification of PSE-like, normal, and DFD-like meat

	L* values	pH values
^≠^PSE-like	L* > 53.00	pH < 5.60
^#^Normal	44.00 ≤ L * ≤ 53.00	5.60 ≤ pH ≤5.90
^#^DFD-like	L * < 44.00	pH > 5.90

^≠^Carvalho et al. [8] and ^#^Carvalho [23]

Color determinations were performed using a Minolta CR-400 colorimeter using five different reading points per sample (L*, a*, b*), as described in Carvalho et al. [2]. The pH was measured in duplicate by inserting electrodes into the breast meat (Testo 205, Testo AG, Lenzkirch, Germany) as described in Carvalho et al. [24].

## Results and discussion

### Truck container microenvironment assessment

**Fig. 3:**
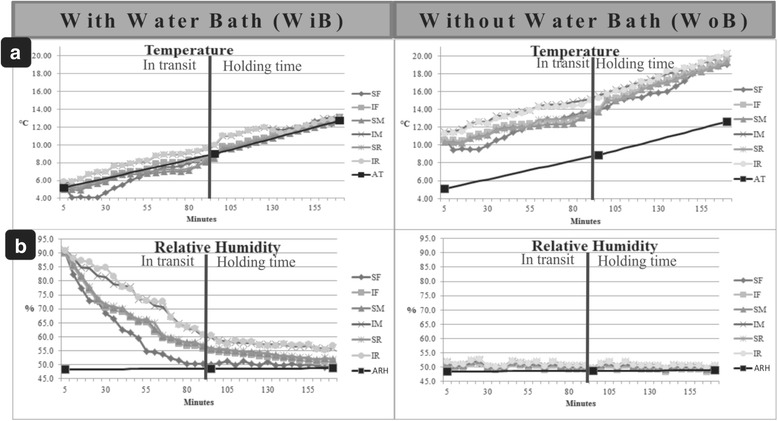
Variations in (**a**) temperature and (**b**) relative humidity (RH) in the container truck with elapsed time for the different turkey transport treatments superior front (SF), inferior front (IF), superior middle (SM), inferior middle (IM), superior rear (SR) and inferior rear (IR) and ambient temperature (AT) and ambient relative humidity (ARH) during a typical winter journey of 95 min and a holding time of 70 min at the slaughterhouse. *n* = 8 per treatment

Table 2 lists the results of T and RH. No significant interactions between bath treatment and truck container compartment position for T (*P* ≥ 0.01) were observed. However, by analyzing separately first the bath and subsequently the birds’ container positions, they indeed were significantly different (*P* < 0.01). Pre-transport water baths caused a temperature drop within the every truck compartment. WoB group temperatures were an average of 7.64 °C higher than WiB group. Positionally, the temperatures of all superior regions of the truck (SF, SM and SR) and the IF position were lower than those in the IM and IR compartments (Table 2). RH is strongly correlated with truck compartment position and bath treatment (*P* < 0.01). The highest RH values were observed for IM and IR in the WiB groups, and the lowest RH values occurred for WoB groups regardless the truck compartment. Figure 3 shows detailed step-by-step variations throughout the journey from the farm to the processing plant and subsequent the holding time for T (A) and RH (B).

**Table 2:** Mean values of the Temperature (T) and the Relative Humidity (RH) determined with (WiB) or without (WoB) bath treatments immediately before leaving the farm. Birds were located in one of 6 different container compartments: superior front (SF), inferior front (IF), superior middle (SM), inferior middle (IM), superior rear (SR) and inferior rear (IR)

	Vehicle compartments	WiB	WoB	Average
^#^T (°C)	SF	6.28	13.93	10.10^b^
	IF	7.06	14.38	10.72^ab^
	SM	6.47	13.97	10.22^b^
	IM	7.91	15.89	11.90^a^
	SR	6.71	14.27	10.49^ab^
	IR	7.99	15.87	11.93^a^
CV = 15.62%	Average	7.07^B^	14.71^A^	
^$^RH (%)	SF	61.77^A,c^	50.07^B,a^	55.92
	IF	66.60^A,c^	49.38^B,a^	57.99
	SM	65.93^A,c^	50.22^B,a^	58.07
	IM	74.39^A,ab^	50.34^B,a^	62.36
	SR	67.77^A,bc^	50.73^B,a^	59.25
	IR	75.25^A,a^	50.66^B,a^	62.95
CV = 11.74%	Average	68.62	50.23	

^a-c^Different letters on the same column indicate significant differences, as measured by the Tukey test (*P* < 0.01). ^A-B^ Different letters on the same line indicate significant differences, as measured by the Tukey test (*P* < 0.01). ^$^ The analyzed variables exhibited interaction to each other (*P* < 0.01). ^#^ The analyzed variables did not exhibit interactions with each other (*P* ≥ 0.01). *n* = 32 transport journeys

In Brazil, currently, there is a pattern for truck container for transport of live turkeys from farms to the commercial slaughterhouses irrespective of the specific season. However, its design does not meet the wide range of tropical and subtropical climates found in this continental size country. Watts et al. [25] reported experiments at temperature of up to 20 °C, when broilers showed low production of heat and relative humidity. Conversely, they found that low temperatures resulted in increased heat production by birds in-transit. In our experiments, the exposure of poultry to a temperature of 5 °C in-transit led to a temperature increase in every container compartment at the end of the journey, suggesting heat production by the birds (Fig. 3B). The application of a pre-transportation bath decreased temperature as reported in previous studies [9–11]. In addition by producing heat in cold temperatures, these birds also retained more moisture [25, 26].

**Fig. 4:**
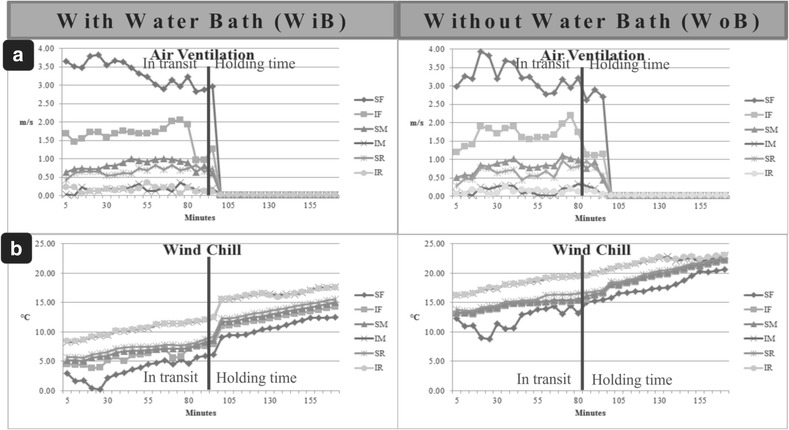
Variations in (**a**) air ventilation and (**b**) wind chill (WC) in the container truck with elapsed time for the different turkey transport treatments during a typical winter journey of 95 min and a holding time of 70 min at the slaughterhouse. n = 8 per treatment

Watts et al. [25] suggested maintenance of environmental conditions during transport in which the birds produce as little as heat possible. Ideally, the temperature should remain within the birds’ thermal neutral zone, i.e.*,* between 23 and 29 °C [27] or 18 and 30 °C [28]. Results herein show the formation of a thermal core at the inferior middle and rear regions of the truck due to heat production by the birds [25] and poorer ventilation within IM and IR compartments (Fig. 3A, Table 3). Therefore, it is concluded that there was no heat dissipation from these regions of the container environment. The IM/IR thermal core also showed higher RH, which is harmful to the welfare of the birds. Watts et al. [25] also stated that effective management of poultry transport must to take into consideration high environment humidity levels that exacerbate the stressful effects on birds. These findings corroborate other reports on bird welfare that stated birds would be able to withstand the unfavorable effects of low temperatures by maintaining a dry environment in-transit [7]. Furthermore, the thermal core found in our study is different in relation to the experiments carried out in other countries [12, 29], because of the truck container design. Hoxey et al. [13] and Mitchell and Kettlewell [29] reported thermal core formation at the front of containers that had an air impermeable barrier at the front of the truck. Conversely, truck containers commonly used in Brazil are fully ventilated, allowing air to enter during vehicle motion, thus promoting more air flow in the front compartments SF and IF (Table 3 and Fig. 4) as previously shown by Spurio et al. [11]. Figure 4 shows in detail step-by-step variations throughout the journey from the farm to the slaughterhouse and during the holding time for AV (A), and WC (B).

**Table 3:** Mean values of the Air Ventilation (AV) and the Wind Chill (WC) measured with (WiB) or without (WoB) bath treatments immediately before leaving the farm. Birds were located in one of 6 different container compartments: superior front (SF), inferior front (IF), superior middle (SM), inferior middle (IM), superior rear (SR) and inferior rear (IR)

	Vehicle compartments	WiB	WoB	Average
^#^AV	SF	3.27	3.15	3.21^a^
	IF	1.61	1.60	1.61^b^
	SM	0.83	0.81	0.82^c^
	IM	0.17	0.15	0.16^d^
	SR	0.67	0.65	0.66^c^
	IR	0.17	0.13	0.15^d^
CV = 24.13%	Average	1.12^A^	1.08^A^	
^#^ WC	SF	3.86	13.16	8.51^c^
	IF	6.16	14.56	10.36^ab^
	SM	6.66	14.89	10.77^ab^
	IM	10.43	18.41	14.42^a^
	SR	7.27	15.45	11.36^ab^
	IR	10.51	18.49	14.50^a^
CV = 23.21%	Average	7.48^B^	15.82^A^	

^a-c^Different letters on the same column indicate significant differences, as measured by the Tukey test (*P* < 0.01). ^A-B^ Different letters on the same line indicate significant differences, as measured by the Tukey test (*P* < 0.01). ^#^ The analyzed variables did not exhibit interactions with each other (*P* ≥ 0.01). n = 32 transport journeys

Table 3 and Fig. 4 present the values obtained for AV (m/s) and WC (°C), which was determined by using the association of air temperature and air ventilation to measure the perceived equivalent temperature felt by the birds [17, 22]. For AV, no interaction (*P* ≥ 0.01) was found between the truck compartment and bath treatment. However, by separately analyzing firstly the AV and subsequently the WC felt by the birds, they indeed were significantly different (*P* < 0.01). Lower average values of AV were observed at the IR and IM positions, regardless of whether the birds were under WiB or WoB treatment. The highest AV value was recorded at the SF position, exhibiting a difference of 3.05 m/s relative to the lowest values at the IM and IR positions.

Similarly to AV, no interaction was observed between the truck compartment and bath treatment for WC (*P* ≥ 0.01), although separately analyzing firstly the bath and subsequently the bird’s container position, they were significantly different (*P* < 0.01). The WiB treatment group showed lower WC values. The animals in the SF presented lower WC values because of the lower T (Table 2) and highest AV (Table 3 and Fig. 4) values. The WC values varied from 3 to 18 °C. The 15 °C WC variation within the truck containers, which incorporated the AV measurements, was smaller than the temperature variation measured outside of the truck containers, which include an AV value in their determination [22].

### Meat quality and meat color abnormalities

The data in Table 4 indicate that for the pH and L* values, significant interactions (*P* < 0.01) occurred between compartments and bath treatment. The highest pH values observed were for IM and IR compartments under WiB. Measured pH values did not differ under WoB regardless of truck compartment. For the L* value, there was an interaction (P < 0.01) between truck compartments and bath treatment (Table 4), with lower values observed for IM and IR under WiB treatment and the highest values observed in WoB, regardless of truck compartment.

**Table 4:** Mean values of pH_24_ h and L* determined with (WiB) or without (WoB) bath treatments immediately before leaving the farm. Birds were located in one of 6 different container compartments: superior front (SF), inferior front (IF), superior middle (SM), inferior middle (IM), superior rear (SR) and inferior rear (IR)

	Vehicle compartments	WiB	WoB	Average
^$^pH_24_	SF	5.79^A,c^	5.81^A,a^	5.80
	IF	5.82^A,c^	5.84^A,a^	5.83
	SM	5.80^A,c^	5.82^A,a^	5.81
	IM	5.89^A,ab^	5.85^B,a^	5.87
	SR	5.84^A,bc^	5.83^A,a^	5.84
	IR	5.92^A,a^	5.85^B,a^	5.89
CV = 35.98%	Average	5.85	5.83	
^$^L	SF	50.27^A,a^	48.99^A,a^	49.63
	IF	48.52^A,ab^	48.24^A,a^	48.38
	SM	48.15^A,b^	48.46^A,a^	48.30
	IM	45.67^B,c^	47.37^A,a^	46.52
	SR	47.81^A,b^	47.49^A,a^	47.65
	IR	44.27^B,c^	47.07^A,a^	45.67
CV = 11.74%	Average	47.45	47.93	

^a-c^Different letters on the same column indicate significant differences, as measured by the Tukey test (*P* < 0.01). ^A-B^ Different letters on the same line indicate significant differences, as measured by the Tukey test (*P* < 0.01). ^$^The analyzed variables exhibited interactions with each other (*P* < 0.01). n = 1344 breast meat samples were shared into transport journeys

Temperatures inside poultry transport vehicles have been intensely studied and related to physiological signs for poor animal welfare and meat quality [30–32]. The ambient temperatures for birds in-transit affect meat quality parameters [11, 16]. Indeed in this experiment, we found differences in meat quality parameters due to the location of truck container compartments (Tables 4 and 5). Various authors have reported that exposure of birds to low temperatures (4, 5, and 7 °C) before slaughter resulted in breast meat with better functional properties because of higher *postmortem* pH values [10, 33–35]. In our case, the highest pH values were observed in IR and IM positions, indicating the importance of higher relative humidity in cold weather conditions affecting the WC while in-transit [25].

**Table 5:** Mean values of the PSE-like and the DFD-like incidence (%) throughout the turkey transportation period with (WiB) or without (WoB) water bath treatments immediately before leaving the farm. Birds were located in one of 6 different truck container compartments: superior front (SF), inferior front (IF), superior middle (SM), inferior middle (IM), superior rear (SR) and inferior rear (IR)

	Vehicle compartments	WiB	WoB	Average
^#^PSE-like (%)	SF	12.0	7.0	9.0^a^
	IF	9.0	6.0	7.0^ab^
	SM	2.0	6.0	4.0^abc^
	IM	1.0	1.0	1.0^c^
	SR	5.0	2.0	4.0^bc^
	IR	1.0	1.0	1.0^c^
CV = 26.51%	Average	5.0^A^	4.0^A^	
^$^DFD-like (%)	SF	4.0^A,c^	3.0^A,a^	3.5
	IF	4.0^A,c^	3.0^A,a^	3.5
	SM	13.0^A,b^	5.0^B,a^	11.5
	IM	28.0^A,a^	6.0^B,a^	17.0
	SR	17.0^A,b^	4.0^B,a^	10.5
	IR	25.0^A,a^	6.0^B,a^	15.5
CV = 32.07%	Average	15.0	4.5	

^a-c^Different letters on the same column indicate significant differences, as measured by the Tukey test (*P* < 0.01). ^A-B^ Different letters on the same line indicate significant differences, as measured by the Tukey test (*P* < 0.01). ^$^The analyzed variables exhibited interactions with each other (*P* < 0.01). ^#^The analyzed variables did not exhibit interactions with each other (*P* ≥ 0.01). n = 1344 breast meat samples were shared into transport journeys

Our results showed that samples taken from IM and IR positions had darker breast meat. Color variation is an important attributed for consumer acceptance, especially if multiple color fillets were packed with noticeable color differences [36]. According to Dadgar et al. [16], cold conditions during transport adversely affect the breast color, and at temperatures below 0 °C the breast meat darkened significantly. Bianchi et al. [37] reported that chicken breast meat exposed to temperatures below 12 °C was significantly darker compared with those exposed to temperatures between 12 °C and 18 °C and above 18 °C. Babji et al. [34] reported that turkeys subjected to cold weather (4 °C for 4 h) and control (21 °C for 4 h) exhibited significantly higher myoglobin content than did turkeys subjected to heat treatment (38 °C) for 4 h.

Watts et al. [25] reported that birds exposed to negative temperatures had depleted energy reserves (glycogen), resulting in serious damage to animal welfare, an increase in DOA, and lower meat quality. These transport outcomes cause a great deal of financial loss for the poultry industry [38]. Another important factor that can reduce meat abnormalities as PSE and DFD in birds is the lairage before slaughter [15, 39, 40], but upon arrival in slaughterhouse, birds of the all groups were placed in lairage area under similar conditions, this fact evidence the impact of transport in birds stress in our study.

Several authors have provided evidence that a system of color and pH measurement tests are important attributes of meat quality, and it is often used as an indicator of PSE and DFD meat [16, 17, 41, 42]. These meats are two of the major quality defects facing the poultry industry due to economic losses. The data in Table 5 indicate that for PSE-like meat incidences, no interaction was found for PSE-like variable (*P* ≥ 0.01) between factors (truck compartment and bath treatment). However, when analyzing factors separately, the effect of truck compartment was significant (*P* < 0.01). Lower PSE-like values were found in meat samples taken equally from animals located at IR and IM, but the highest PSE-like meat incidences were found at the front of the truck compartment independent use of bath*.*

In testing for DFD-like meat, there was an interaction (*P* < 0.01) between factors (truck compartment and bath treatment). The highest DFD-like value was found in meat samples taken equally from animals at IR and IM compartments under WiB treatment. The lowest DFD-like meat incidence was found in birds located at every compartment of WoB group.

The highest incidence of DFD-like meat was found at the IM and IR compartments. Associating these results with the microclimate data (T, RH and AV) suggests that WC directly affected animal welfare; low-temperature values combined with high wind velocity caused thermal discomfort [16, 17, 22]. Such hypothermal conditions cause depletion of muscle glycogen in order to keep the body warm. Basal metabolic activities are amplified in response to temperatures below the comfort zone in order to maintain body temperature in homoeothermic species [17, 43]. In addition, our results indicate high RH at the rear truck container leading to higher incidence of DFD-like meat, showing the relationship between RH, animal welfare, and thus meat quality.

Turkey breast meat harvested during the winter in Brazil had higher pH values and lower L * values than those for meat harvested during the summer [8]. In our experiments, the environmental temperature of 5 °C and high relative humidity (Table 2) at IR and IM positions promoted the formation of DFD-like meat, while in the SF, IF and SM compartments, there was a higher incidence of PSE-like meat. These findings led us to believe that two phenomena that impact animal welfare occurred simultaneously: hypothermia and hyperthermia. DFD-like meat was associated with hypothermia as the birds exposed to a wet environment with excessive RH and relatively low T, formed an unfavorable thermal core at the IR and IM compartments. Consequently, the birds used their reserved energy (glycogen) to maintain thermal homeostasis [16, 17, 43, 44] resulting in DFD meat [16, 17, 45]. The physiological stress leading to PSE-like meat formation can be closely associated to mammal malignant hyperthermia (MH), which is triggered by genetic factors [46]. The MH is responsible for the series of biochemical reactions resulting in uncontrollable glycolysis with increased lactic acid production because of the excessive Ca^2+^ within the sarcoplasm milieu [47]. In our study, the frontal compartments showed a high-ventilation microenvironment in cold weather, resulting in homeostatic imbalance in the poultry for a short period, inducing the formation of PSE-like meat.

The first priority is to introduce management tools as to maintain animal welfare and thus prevent stressful conditions for the birds. Tools as protect birds from wind and rain during winter transport through improving the aerodynamics and design of the truck.

## Conclusion

The WC and RH were important factors on the turkey’s welfare in winter season. The results demonstrate that for birds transported under these conditions the breast meat quality was affected, bringing about variations in meat color abnormalities. Thus, a better truck container design is necessary to maintain an adequate microenvironment throughout the vehicle.

## Abbreviations

AV: Air ventilation
DFD: Dark, firm and dry
IF: Inferior front
IM: Inferior middle
IR: Inferior rear
PSE: Pale, soft and exudative
RH: Relative humidity
SF: Superior front
SM: Superior middle
SR: Superior rear
T: Temperature
WC: Wind chill.
WiB: With water bath
WoB: Without water bath
