# Correction to: in-transit development of color abnormalities in turkey breast meat during winter season

**DOI:** 10.1186/s40781-018-0162-z

**Published:** 2018-02-16

**Authors:** Rafael H. Carvalho, Danielle C. B. Honorato, Paulo D. Guarnieri, Adriana L. Soares, Mayka R. Pedrão, Alexandre Oba, Fernanda G. Paião, Elza I. Ida, Massami Shimokomaki

**Affiliations:** 1 Graduate Program in Animal Science, Department of Veterinary Preventive Medicine, Londrina State University, PO Box 6001, Londrina, PR CEP 86010–951 Brazil; 2 Graduate Program in Food Science, Department of Food Science and Technology, Londrina State University, Londrina, PR Brazil; 3 Graduate Program in Food Science, Sao Paulo University, Sao Paulo, SP Brazil; 4 Professional Master Program, Paraná Federal Technological University in Londrina, Campus Londrina, Londrina, PR Brazil

## Correction

Due to a technical issue this article [1] was accidentally published in volume 59, the correct volume for this article is volume 60.
